# Open questions on the photophysics of thermally activated delayed fluorescence

**DOI:** 10.1038/s42004-021-00533-y

**Published:** 2021-06-17

**Authors:** Julien Eng, Thomas J. Penfold

**Affiliations:** Chemistry, School of Natural and Environmental Sciences, Newcastle University, Newcastle Upon Tyne, UK

**Keywords:** Light harvesting, Materials for devices

## Abstract

The process of thermally activated delayed fluorescence (TADF) converts non-radiative triplet states into emissive singlet states. Herein we outline the fundamentals of TADF, some of the recent progress in understanding the key material properties responsible for promoting TADF and finally discuss some remaining challenges for the  potential applications of this phenomenon.

Thermally Activated Delayed Fluorescence (TADF), originally called E-type delayed fluorescence, is defined as the fluorescence occurring from an excited state which has previously populated a triplet state. Consequently, it is not observed during  photophysical investigations of many organic molecules as: i) the rate of intersystem has to be greater than the rate of fluorescence and ii) the energy gap between singlet and triplet state needs to be <0.5 eV. Indeed, due to its often minor contribution to the overall photocycle of many excited molecules, for a long time it remained a largely academic curiosity. However, TADF has recently gained significant attention in the context of organic electronics owing to its ability to transform often detrimental triplets states into emissive singlet states^1^. In addition, it can give rise to long fluorescence lifetimes ranging from nanoseconds to milliseconds, which offers new strategies for time-resolved luminescence imaging and sensing^2^. It has also been explored in the context of photocatalysis^3^ and sensors^4^.

Originally, TADF was described within the framework of a thermal equilibrium between the lowest singlet and triplet excited states, which is based upon the assumption that the rates of intersystem (k_*ISC*_) and reverse intersystem (k_*rISC*_) crossing are fast compared to the fluorescence rate (k_*F*_). Within this framework, the rate of TADF (k_*TADF*_) is defined:

1 $${k}_{TADF}=\frac{1}{3}{k}_{F}\exp \left[\frac{-{{\Delta }}{E}_{ST}}{{k}_{B}T}\right]$$

where *k*_*B*_ is the Boltzmann constant and *T* the temperature (Fig. 1a). This Arrhenius type behaviour emphasises the importance of a small energy gap (Δ*E*_*ST*_) between the active singlet and triplet states. This gap is usually minimised using charge transfer states. The presence of charge transfer (CT) states is typically ensured by the use  a Donor-Acceptor (D-A) framework where an electron-donating moiety is linked to an electron-accepting moiety via a molecular spacer. However this assumes that the states involved are of the same character and therefore spin-orbit coupling (SOC) between them is formally forbidden^5^. Without this coupling questions arise about how the different spin states couple and therefore TADF operates.

**Fig. 1:**
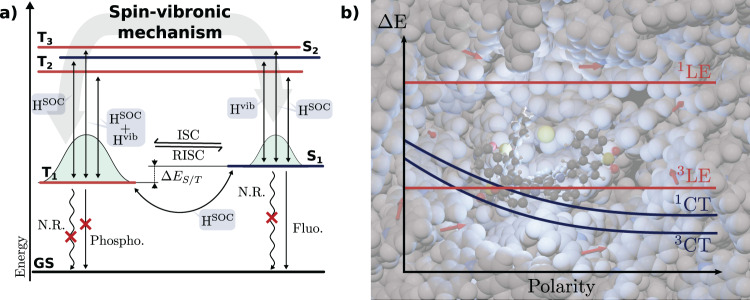
Spin-vibronic mechanism. **a** Schematic representation of the TADF mechanism. H^SOC^ is the spin-orbit coupling. H^vib^ is the vibronic coupling. N.R. Non-Radiative decay pathways, Fluo. Fluorescence, Phospho. Phosphorescence. **b** Effect of the polarity of the environment on the energy of excited state involved in the TADF process. The locally excited (LE), e.g. ππ^*^, state is much less influenced by host polarity than the charge transfer (CT) states which have a large dipole moment.

This question and the increased interest in TADF has illustrated the importance of a detailed understanding of the excited state mechanism involved, especially from the perspective of designing new materials. In this comment, we discuss recent findings on the mechanism for TADF, present some open questions for the future of TADF, and consider how these studies could be transferred from fundamental to applied science.

## Mechanism of (reverse) intersystem crossing

The rate of reverse intersystem crossing (rISC) between a triplet and singlet state can be described using Fermi’s Golden Rule^5^:

2 $${k}_{rISC}=\frac{2\pi \rho }{Z\hslash }| M{| }^{2},$$

where *M* is the coupling between initial and final states driven by the SOC, *Z* is the canonical partition function and *ρ* is associated with the vibrational density of states. This highlights the variables, electronic and vibrational, which can be modified to manipulate rISC^5^. The most obvious is the electronic term, namely SOC which couples the two states. In the context of purely organic TADF molecules, the absence of the heavy elements places an upper limit of how large SOC can be, which can be somewhat overcome using heavier elements^6–8^. But even in these cases, it remains limited as the energy gap which is minimised during the design of TADF emitters usually relies on singlet and triplet states which are of same character. In this case, SOC between these two states is forbidden as the change of spin multiplicity cannot be accompanied by a change of angular momentum, independent of the presence or not of heavy elements^5^. Consequently, electronic states of a different nature (e.g. locally excited (LE) states such as a ππ^*^ state) close in energy to the CT singlet/triplet pair is required to promote coupling between the two states via the spin-vibronic mechanism^9^. Conformational dynamics, often dominated by a rotation around the D-A bond, promotes this spin- vibronic mixing (Fig. 1a) between these close-lying singlet and triplet states so that they are then no longer exactly of the same nature, permitting SOC.

The presence of multiple states of different character gives rise to the importance of the embedding environment around the TADF emitter, as shown schematically in Fig. 1b. Indeed, the large dipole moment associated with a CT state induces strong interactions with local environment which increase with polarity. As the same shifts do not occur, due to an absence of a dipole moment, for LE states which participate in the TADF mechanism (See Fig. 1b), the relative energy gap and therefore mixing between these state will strongly depend of the host environment. Consequently, guest-host interactions can play a critical role in TADF. The properties of the environment, including dielectric properties, mobility, viscosity have been shown to greatly influence the relative energies and mixing of excited states involved in the TADF process^10^. Recently, Dhali et al.^11^ have developed a model which simultaneously incorporates the concurrent role played by conformational degrees of freedom and environment effects. This established a clear understanding and illustrates the huge progress made in this area, but also highlights the large number of interconnected properties that one must consider when understanding TADF.

## Achieving fine control of excited-state dynamics

The importance of conformational dynamics highlights a key challenge for understanding and controlling the properties of molecules exhibiting TADF. Indeed, the conformational dynamics which contribute the TADF mechanism may also be responsible for undesirable processes such as non-radiative decay. Indeed, in the context of D-A TADF molecules, near orthogonality between D-A units is required to minimise the energy gap between the singlet and triplet states. But simultaneously, vibrations away from orthogonality are required to promote rISC and increase the radiative rate of the charge transfer states involved in the emission. Constraining the dihedral bond too rigidly, e.g. to reduce non-radiative decay, is detrimental as it prevents rotational/breathing motions important for the vibrational coupling mechanism, and tends to yield room temperature phosphorescence^12^. Consequently, using explicit covalent bonds to constrain the D-A angle are often too restrictive and therefore recent work has focused upon covalent modifications, such as substitution to enhance steric hindrance^13,14^. Alternatively, Rajamalli et al.^15^ recently developed a rotaxane based upon a carbazole-benzophenone TADF emitter. They demonstrated that non-covalent interactions between the TADF emitter and macrocycle forming the mechanical bond enforced subtle changes in the conformational dynamics responsible for improvements in key photophysical properties, notably the photoluminescence quantum yield and a decrease in the energy difference between singlet and triplet states, as well as fine tuning of the emission wavelength.

Such interactions could also be exploited to reduce heterogeneity and dispersive rISC rates^16^, a key objective towards maximising rISC rates. Recently, dos Santos et al.^17^ proposed a D-A_3_ strategy, based upon a rigid triazatruxene donor core with three dibenzothiophene-S,S-dioxide peripheral acceptors. The multiple donor-acceptor interactions yield a high density of excited states, which give a significant enhancement to the rISC rate, leading to delayed fluorescence decay times as low as ~100 ns. However, subtle excited state conformational dynamics reduces the advantageous high density of state, causing the rISC rate to slow down making it similar to typical D-A TADF systems^18^. This is because the excited state potential generates an inequivalence in the D-A bond lengths and the lowest excited states are associated with the largest D-A bond length. Consequently, the structural relaxation generates an energetic landscape similar to D-A molecules. In this scenario, steric hindrance and non-covalent interactions could be used to deliver fine control of the excited state dynamics reducing these conformational changes and increasing rISC rates.

## Multi-resonance approach to TADF

**Fig. 2:**
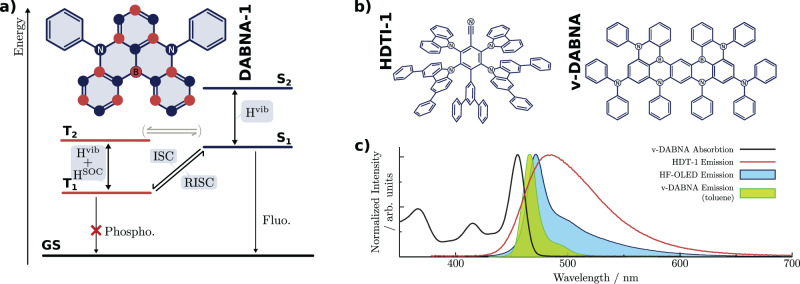
Multi-resonance TADF Emitters. **a** TADF mechanism for the MR-TADF molecule DABNA^23^, involving vibronic coupling between multiple excited states similar to the D-A framework. The resonance effect is shown inset with the difference of density associated with the S_1_ ← GS (T_1_ ← GS) difference of electronic density: gain of electronic density in blue, loss in red. ISC Intersystem Crossing, RISC Reverse ISC, H^vib^ Vibronic coupling, H^SOC^ Spin-orbit coupling. **b** Structure of the HDTI-1 and v-DABNA used in the hyperfluorescence OLED presented in^21^. **c** Absorption of v-DABNA (full black lines)^21^, emission of HDTI-1 (full red lines)^21^, emission of the hyperfluorescence OLED device (blue line and blue area)^21^ and emission of v-DABNA^25^ (full green lines and green area).

Reducing the exchange energy between the unpaired electrons to minimise the gap between singlet and triplet states has predominantly been achieved using CT states within D-A framework. A highly appealing alternative approach is the so-called multi-resonance-based TADF (MR-TADF) materials, which are based upon on p- and n-doped polycyclic aromatic hydrocarbons (Fig. 2a). These exploit spatial symmetry (resonance) to separate the frontier orbitals involved. While the initial MR-TADF molecules developed^19^ still retained a relatively large energy gap (0.2 eV), by expansion of the *π*-framework an energy gap of <0.1 eV has been achieved^20^ and this leads to an increase in the rISC rate to ~10^5^ s^−1^, analogous to many other TADF emitters based upon the D-A framework. These emitters have recently been exploited in the 3^rd^ generation of OLEDs and those based upon Hyperfluorescence (Fig. 2b)^21^. Besides the energy gap, the use of only carbon, nitrogen and boron means that the SOC in MR-TADF type molecules is small. Recently by incorporating sulfur into the framework, Hua et al.^22^ have demonstrated it is possible to increase the rate of rISC by over an order of magnitude.

Despite the very different nature of their design, theoretical^23,24^ and experimental^25^ studies have shown that like D-A TADF emitters, the MR-TADF emitters operate via vibrationally enhanced rISC, and this theme continues within other TADF frameworks^26,27^.

## Open questions and outlook

Through an intense research effort, significant progress has been made in understanding TADF. Core design considerations and multiple architectures have been developed and these extensive works have demonstrated that TADF is applicable in both fundamental and applied areas including light emission, photoredox catalysis, solar energy conversion, and excited-state decay engineering^28^. However, to improve and optimise properties, it is clear that design needs to simultaneously consider the concurrent role of electronic structure, conformational degrees of freedom and environmental effects. In addition, going beyond understanding and demonstrating the ability to control and manipulate at will the properties of TADF is a crucial next stage. In this direction, some rational design approaches have begun to appear, with promising results^29^.

Key from a theoretical perspective is how we transform understanding to design. Models developed to understand the nature of TADF can be tuned to optimise performance^30^, but these optimisations subsequently need to be transformed in the chemical structures. Sophisticated high-throughput approaches have been proposed^31^, although on the scale these operated, they were unable to go beyond simulating the energy gap and oscillator strength which cannot provide the full picture. Recently Zhao et al.^32^ implemented a hierarchical approach in the sense that as materials were ruled out, the remaining molecules were tested using a high level of theory. It is likely that combining all of these approaches will be required to expand and then subsequently refine the chemical space to identify high-performance materials.

Owing to its Boltzmann behaviour, one of the biggest limitations for TADF materials is the rate of the thermally activated transfer from the triplet and singlet state. Small energy gaps facilitate thermal upconversion, however recently a number of molecules have been introduced which invert the relative energy between first excited triplet and singlet states, i.e. break Hund’s first rule, with the potential to significantly enhance triplet harvesting^33^. Breaking Hund’s first rule is rare and therefore to date only molecules based around a heptaazaphenalene (TAHz)^34^ core have been proposed, but when systematically explored and exploited these observations could have profound implications.

## References

[1] Uoyama H, Goushi K, Shizu K, Nomura H, Adachi C (2012). Highly efficient organic light-emitting diodes from delayed fluorescence. Nature.

[2] Ni F, Li N, Zhan L, Yang C (2020). Organic thermally activated delayed fluorescence materials for time-resolved luminescence imaging and sensing. Adv. Opt. Mater..

[3] Speckmeier E, Fischer TG, K Z (2018). A toolbox approach to construct broadly applicable metal-free catalysts for photoredox chemistry: Deliberate tuning of redox potentials and importance of halogens in donor-acceptor cyanoarenes. J. Am. Chem. Soc..

[4] Lee YH, Jana S, Lee H, Lee SU, Lee MH (2018). Rational design of time-resolved turn-on fluorescence sensors: exploiting delayed fluorescence for hydrogen peroxide sensing. Chem. Commun..

[5] Penfold TJ, Gindensperger E, Daniel C, Marian CM (2018). Spin-vibronic mechanism for intersystem crossing. Chem. Rev..

[6] Hamze R (2019). Eliminating nonradiative decay in cu(i) emitters: >99% quantum efficiency and microsecond lifetime. Science.

[7] Pander P (2021). Exceptionally fast radiative decay of a dinuclear platinum complex through thermally activated delayed fluorescence. Chem. Sci..

[8] Zhang Y (2020). Delayed fluorescence from a zirconium(iv) photosensitizer with ligand-to-metal charge-transfer excited states. Nat. Chem.

[9] Gibson J, Monkman AP, Penfold TJ (2016). The importance of vibronic coupling for efficient reverse intersystem crossing in thermally activated delayed fluorescence molecules. ChemPhysChem.

[10] Northey T, Stacey J, Penfold TJ (2017). The role of solid state solvation on the charge transfer state of a thermally activated delayed fluorescence emitter. J. Mater. Chem. C.

[11] Dhali R (2021). Understanding TADF: a joint experimental and theoretical study of DMAC-TRZ. Phys. Chem. Chem. Phys..

[12] Ward JS (2018). Bond rotations and heteroatom effects in donor–acceptor–donor molecules: Implications for thermally activated delayed fluorescence and room temperature phosphorescence. J. Org. Chem..

[13] Park IS, Lee J, Yasuda T (2016). High-performance blue organic light-emitting diodes with 20% external electroluminescence quantum efficiency based on pyrimidine-containing thermally activated delayed fluorescence emitters. J. Mater. Chem. C.

[14] Chen X-K (2019). Intramolecular noncovalent interactions facilitate thermally activated delayed fluorescence (TADF). J. Phys. Chem. Lett..

[15] Rajamalli P (2021). Using the mechanical bond to tune the performance of a thermally activated delayed fluorescence emitter. Angew. Chem. Int. Ed..

[16] Serevičius T (2019). Emission wavelength dependence on the rISC rate in TADF compounds with large conformational disorder. Chem. Commun..

[17] dosSantos PL (2018). Triazatruxene: A rigid central donor unit for a d-a3 thermally activated delayed fluorescence material exhibiting sub-microsecond reverse intersystem crossing and unity quantum yield via multiple singlet-triplet state pairs. Adv. Sci.

[18] Eng J, Hagon J, Penfold TJD (2019). D–a3 TADF emitters: the role of the density of states for achieving faster triplet harvesting rates. J. Mater. Chem. C.

[19] Hatakeyama T (2016). Ultrapure blue thermally activated delayed fluorescence molecules: Efficient HOMO-LUMO separation by the multiple resonance effect. Adv. Mater..

[20] Kondo Y (2019). Narrowband deep-blue organic light-emitting diode featuring an organoboron-based emitter. Nat. Photonics.

[21] Chan C-Y (2021). Stable pure-blue hyperfluorescence organic light-emitting diodes with high-efficiency and narrow emission. Nat. Photonics.

[22] Hua, T.et al. Heavy-atom effect promotes multi-resonance thermally activated delayed fluorescence. *ChemRxiv*10.26434/chemrxiv.14046296.v1 (2021).

[23] Northey T, Penfold TJ (2018). The intersystem crossing mechanism of an ultrapure blue organoboron emitter. Org. Electron.

[24] Kim, I.et al. Three-state-involving vibronic resonance is a key to enhancing reverse intersystem crossing dynamics of organoboron-based ultrapure blue emitters. *ChemRxiv*10.26434/chemrxiv.12413417.v1 (2020). 10.1021/jacsau.1c00179 PMC8395647 34467345

[25] Stavrou K, Danos A, Hama T, Hatakeyama T, Monkman AP (2021). Hot vibrational states in a high-performance multiple resonance emitter and the effect of excimer quenching on organic light-emitting diodes. ACS Appl. Mater. Interfaces.

[26] Cao Y, Eng J, Penfold TJ (2019). Excited state intramolecular proton transfer dynamics for triplet harvesting in organic molecules. J. Phys. Chem. A.

[27] Long Y (2020). Excited state dynamics of thermally activated delayed fluorescence from an excited state intramolecular proton transfer system. J. Phys. Chem. Lett..

[28] Yonemoto DT, Papa CM, Mongin C, Castellano FN (2020). Thermally activated delayed photoluminescence: deterministic control of excited-state decay. J. Am. Chem. Soc..

[29] Wada Y, Nakagawa H, Matsumoto S, Wakisaka Y, Kaji H (2020). Organic light emitters exhibiting very fast reverse intersystem crossing. Nat. Photonics.

[30] de Silva P, Kim CA, Zhu T, Van Voorhis T (2019). Extracting design principles for efficient thermally activated delayed fluorescence (TADF) from a simple four-state model. Chem. Mater..

[31] Gómez-Bombarelli R (2016). Design of efficient molecular organic light-emitting diodes by a high-throughput virtual screening and experimental approach. Nat. Mater..

[32] Zhao K, Omar ÖH, Nematiaram T, Padula D, Troisi A (2021). Novel thermally activated delayed fluorescence materials by high-throughput virtual screening: going beyond donor–acceptor design. J. Mater. Chem. C.

[33] Pollice R, Friederich P, Lavigne C, dosPassosGomes G, Aspuru-Guzik A (2021). Organic molecules with inverted gaps between first excited singlet and triplet states and appreciable fluorescence rates. Matter.

[34] Ehrmaier J (2019). Singlet-triplet inversion in heptazine and in polymeric carbon nitrides. J. Phys. Chem. A.

